# A 4-year-old Boy Positive for Anti-rabphilin-3A Antibody and Diagnosed With Lymphocytic Infundibuloneurohypophysitis

**DOI:** 10.1210/jcemcr/luae214

**Published:** 2024-12-26

**Authors:** Akiko Yamamoto, Nagisa Komatsu, Naoko Iwata, Haruki Fujisawa, Atsushi Suzuki, Yoshihisa Sugimura

**Affiliations:** Division of Pediatrics, Kumamoto Chuo Hospital, Kumamoto 862-0965, Japan; Division of Pediatrics, Kumamoto Chuo Hospital, Kumamoto 862-0965, Japan; Department of Endocrinology and Diabetes, Daido Hospital, Nagoya, Aichi 457-8511, Japan; Department of Endocrinology, Diabetes and Metabolism, School of Medicine, Fujita Health University, Aichi 470-1192, Japan; Department of Endocrinology, Diabetes and Metabolism, School of Medicine, Fujita Health University, Aichi 470-1192, Japan; Department of Endocrinology, Diabetes and Metabolism, School of Medicine, Fujita Health University, Aichi 470-1192, Japan; Department of Endocrinology, Diabetes and Metabolism, School of Medicine, Fujita Health University, Aichi 470-1192, Japan

**Keywords:** anti-rabphilin-3A antibody, arginine vasopressin deficiency (AVP-D), lymphocytic infundibuloneurohypophysitis (LINH)

## Abstract

Lymphocytic infundibuloneurohypophysitis (LINH) is a disease with an etiology involving an autoimmune mechanism, characterized by lymphocytic inflammation of the posterior pituitary and infundibular stalk, resulting in arginine vasopressin deficiency. It is difficult to distinguish from pituitary neoplasm or infiltrative diseases, and biopsy is necessary for a definitive diagnosis, but this is highly invasive. In children, it is especially important to distinguish LINH from tumors such as germ cell tumors. Recently, the usefulness of anti-rabphilin-3A antibody as a serum marker for LINH has been reported. To date, only a limited number of pediatric cases have been reported. We present a 4-year-old boy with arginine vasopressin deficiency. Magnetic resonance imaging of the head showed thickening of the pituitary stalk without a posterior pituitary bright spot, and anti-rabphilin-3A antibody was positive. Consequently, pituitary biopsy was not performed because of the strong suspicion of LINH. Five months after symptom onset, the pituitary stalk thickening had resolved. This case represents the first report of probable or definitive LINH with anti-rabphilin-3A antibody positivity in a 4-year-old child, making it the youngest positive case reported to date. Our case highlights the importance of noninvasive approaches and careful follow-up to avoid invasive interventions for children with LINH.

## Introduction

Thickening of the pituitary stalk is relatively rare in children [1]. The most common causes are germ cell tumors, Langerhans cell histiocytosis, and lymphocytic hypophysitis [2] [3], which are often difficult to differentiate based on clinical symptoms, tumor markers, and imaging findings. Lymphocytic infundibuloneurohypophysitis (LINH), a subtype of lymphocytic hypophysitis, is a disease in which an autoimmune mechanism causes cellular infiltration, mainly involving lymphocytes, into the posterior pituitary and hypothalamic infundibulum area. This results in arginine vasopressin deficiency (AVP-D), known as central diabetes insipidus. LINH was first proposed by Imura et al in 1993 [4]. Anti-rabphilin-3A antibody was initially reported in 2015 [5] as a highly sensitive and specific serum marker for LINH. Since then, many cases of adult LINH positive for anti-rabphilin-3A antibody have been reported. However, there have been only scattered reports of anti-rabphilin-3A antibody in pediatric LINH.

In the present study, we diagnosed a 4-year-old boy with AVP-D, and magnetic resonance imaging (MRI) of the head showed thickening of the pituitary stalk and loss of a pituitary bright spot in the posterior lobe. Because various tumor markers were negative and anti-rabphilin-3A antibody was positive, we suspected LINH and followed up the patient conservatively. This is a report of the youngest patient with LINH who tested positive for anti-rabphilin-3A antibody. The present case highlights the fact that anti-rabphilin-3A antibody is a useful tool for investigating the cause of AVP-D in children and may make the diagnosis of LINH more certain without the need for invasive pituitary biopsy.

## Case Presentation

The patient was 4 years and 3 months old boy. He initially consulted our hospital with chief complaints of polyuria and recurrence of nocturnal enuresis. One month prior to that, he developed thirst and showed increased water consumption, with a marked increase in night-time drinking behavior.

The total urine output measured at home ranged from 4833 to 5800 mL/m^2^ body surface area. He was unable to eat adequately because of severe polydipsia and his weight remained low. There were no perinatal abnormalities, and no pertinent family history was noted.

## Diagnostic Assessment

At the time of his first visit to our clinic (age 4 years and 3 months), he was 98.1 cm (−0.80 SD) in height, 13.15 kg (−1.31 SD) in weight, and his body mass index was 13.66, showing signs of emaciation. Growth curves revealed that his weight had not increased since 3 years and 7 months of age. Physical examination identified a dry oral cavity and abdominal distension resulting from fluid retention.

**Figure 1.**
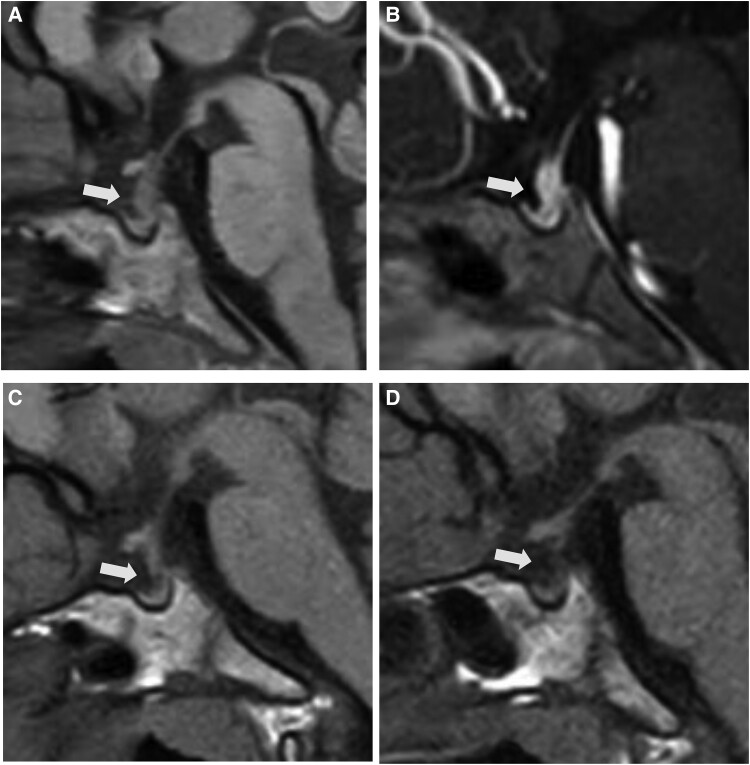
MRI 1 month after the onset of symptoms of uropathy (A) showed loss of the posterior pituitary bright spot and findings of pituitary stalk thickening; contrast-enhanced MRI 2 months later (B) showed slightly progressive swelling of the pituitary stalk, but MRI 4 months after the onset of symptoms confirmed disappearance of the thickening of the pituitary stalk (C). MRI findings at 5 years of age showed a further trend of improvement in pituitary enlargement (D).

Laboratory tests revealed no dehydration or obvious electrolyte abnormalities, serum osmolality within the normal range, no abnormal glucose tolerance, a normal thyroid function, and IGF-1 within the normal age range. Urine specific gravity (1.003) and urine osmolality (85.3 mmol/kg [85.3 mOsm/kg]) (normal reference range: 50-1300 mmol/kg [mOsm/kg]) were both low, with low antidiuretic hormone levels (0.83 pmol/L [0.9 pg/mL]) (normal reference range: <2.58 pmol/L [<2.8 pg/mL]) measured simultaneously. A water deprivation test was performed (Table 1), and when the bodyweight decreased by 3%, hypernatremia and increased serum osmolality were observed, but urinary osmolality remained low and antidiuretic hormone was low relative to serum osmolality, consistent with an arginine vasopressin abnormality. Urinary osmolality increased with vasopressin loading. T1-weighted imaging MRI of the head showed loss of the bright spot in the posterior pituitary gland and thickening of the pituitary stalk (Fig. 1A), leading to a diagnosis of AVP-D. Contrast-enhanced MRI was performed for the thickened pituitary stalk, and the pituitary gland exhibited diffuse enhancement in the early phase, with no obvious areas of poor contrast in the pituitary/pituitary stalk (Fig. 1B).

**Table 1.** Water deprivation test

Time (h)	0	1	2	1 h after desmopressin	2	3	Reference range for basal value
Body weight	13.4 kg	13.2 kg	13.0 kg	12.8 kg	12.6 kg	12.6 kg	
Urine		200 mL	120 mL	120-130 mL	6 mL	6 mL	
Urine specific gravity	1.002	1.002	1.002		1.012	1.011	
Serum Na	145 mmol/L (145 mEq/L)		149 mmol/L (149 mEq/L)			150 mEq/L (150 mmol/L)	136-144 mmol/L (136-144 mEq/L)
Serum osmolality	288 mmol/kg (288 mOsm/kg)		295 mmol/kg (295 mOsm/kg)			297 mmol/kg (297 mOsm/kg)	275-290 mmol/kg (275-290 mOsm/kg)
Urine osmolality	99 mmol/kg (99 mOsm/kg)	120 mmol/kg (120 mOsm/kg)	133 mmol/kg (133 mOsm/kg)	167 mmol/kg (167 mOsm/kg)	471 mmol/kg (471 mOsm/kg)	453 mmol/kg (453 mOsm/kg)	50-1300 mmol/kg (50-1300 mOsm/kg)
ADH	<0.4		0.37 pmol/l (0.4 pg/ml)			6.46 pmol/l (7.0 pg/ml)	<2.58 pmol/l (<2.8 pg/ml)

Abbreviation: ADH, antidiuretic hormone.

Arginine, thyroid-releasing hormone, and corticotropin-releasing hormone loading tests showed a normal anterior pituitary function. Blood α-fetoprotein and β-humanchorionicgonadotropin were both negative, a cerebrospinal fluid examination revealed no increase in the cell count and cerebrospinal fluid placental alkaline phosphatase as a marker for germ cell carcinoma was negative. Anti-rabphilin-3A antibodies, evaluated by western blotting as previously reported, were detected in the patient's serum [5] [6]. Therefore, we decided not to perform pituitary biopsy and follow-up the patient with periodic MRI of the head.

## Treatment

The patient was treated with oral desmopressin: desmopressin acetate hydrate, twice per day to control the urinary output.

## Outcome and Follow-up

After discharge from the hospital, the patient’s desmopressin acetate hydrate was increased according to the urine output. Five months after the onset of symptoms, MRI of the head was performed again, showing that thickening of the pituitary stalk had resolved, although the posterior pituitary bright spot remained absent (Fig. 1C). Based on the imaging, a diagnosis of LINH was made. MRI findings at age 5 years showed a further trend of improvement in pituitary enlargement, and any pituitary bright spot in the posterior lobe continued to remain absent (Fig. 1D). There was no growth rate stagnation and the anterior pituitary function was preserved. We plan to continue patient follow-up to assess the anterior pituitary function while monitoring growth and periodically checking for changes on MRI of the head.

## Discussion

Lymphocytic hypophysitis is a chronic inflammatory condition in which the pituitary gland and hypothalamic stalk are infiltrated with lymphocytes consisting of T, B, and plasma cells. Lymphocytic infiltration and pituitary enlargement are characteristic findings in the early stages of the disease. The disease is classified into 3 categories according to the primary site of involvement: lymphocytic anterior hypophysitis, LINH, and lymphocytic panhypopituitaritis (LPH).

It is important to distinguish it from other neoplastic diseases such as germ cell tumors based on clinical symptoms, tumor markers, and imaging findings, but this is often difficult. Although the exact cause is unknown, autoimmune mechanisms are considered to be involved, and the disease may occur in association with chronic thyroiditis, hypoadrenocorticism, systemic lupus erythematosus, and other conditions [7].

Lymphocytic hypophysitis can be difficult to differentiate from embryonal tumors and other neoplastic diseases because the clinical presentation and imaging findings are often similar, and various tumor markers in blood and spinal fluid are not sensitive. However, distinction between the 2 is important because they are associated with very different treatment strategies and life expectancies. LINH presenting with AVP-D is a lesion confined to the posterior pituitary and neurohypophysis, and the anterior pituitary function is often preserved. MRI shows thickening of the pituitary stalk and loss of a posterior pituitary bright spot. Over time, loss of the posterior pituitary bright spot does not recover, but thickening of the pituitary pattern often resolves, which is a feature of this disease. A definitive diagnosis requires a pituitary biopsy, which shows infiltration of lymphocytes and plasma cells in the pituitary stalk and posterior pituitary lobe. However, pituitary biopsies are rarely performed because of their invasiveness and the risk of inducing hypopituitarism. The average patient age at the onset of LINH is reportedly 42 years [8], and cases involving children are considered rare [9]. However, this may be an underestimate because pituitary biopsies are more difficult and challenging to perform in children than in adults.

At first, anti-rabphilin-3A antibody was reported to be highly sensitive, with a sensitivity of 100% in biopsy-confirmed LINH patients and 76% in clinically diagnosed LINH patients [5], indicating that the test has high-level clinical utility in determining the etiology of AVP-D [6]. Recently, the sensitivity and specificity of anti-rabphilin-3A antibody, where a histological diagnosis was made, was summarized in a recent publication [10]. These cases revealed a sensitivity of LINH was 100.0%, 80.0% for LPH causing AVP-D, and 88.9% for LINH and LPH causing AVP-D; specificity was 97.4% for distinguishing sellar/suprasellar masses. Thus, we believe that anti-rabphilin-3A antibody may be a useful, noninvasive diagnostic marker not only for LINH but also for lymphocytic hypophysitis in general; when LINH is suspected, an anti-rabphilin-3A antibody test should be performed before proceeding to biopsy. Of course, a definitive diagnosis cannot be made without biopsy, and long-term follow-up should be performed in children while considering the possibility of neoplasms.

Pediatric cases reported to date include: a boy with LINH who was found to be positive for anti-rabphilin-3A antibody 9 years after the onset of AVP-D at the age 10 years [11], a 7-year-old LINH patient [12], and 2 children with LPH and lymphocytic anterior hypophysitis (ages 12 and 13 years, respectively) [10], but the number of reports is still small. The present patient was diagnosed at 4 years and 3 months of age, making it the youngest pediatric case reported to date.

An important aspect of diagnosing LINH is the fact that other diseases cannot be completely ruled out without a pituitary biopsy [5]. In 2022, a patient with a pathologically diagnosed germ cell tumor and positivity for anti-rabphilin-3A antibody was reported [10]. Therefore, continued careful monitoring will be necessary in this case.

Furthermore, cautious follow-up is needed in patients with idiopathic AVP-D who test negative for anti-rabphilin-3A antibody. A recent case report including 2 young patients with idiopathic AVP-D who tested negative for anti-rabphilin-3A antibody and were subsequently found to have a germinoma 1.5 years later [13].

Thickening of the pituitary stalk, which disappears with time, may be the basis for the diagnosis of LINH, but the change may be rapid in children. In this case, too, MRI 1 month after the onset of symptoms of enuresis showed thickening of the pituitary stalk, and 2 months later, the stalk was slightly enlarged, but MRI 5 months after symptom onset showed that the thickening had disappeared. It is difficult to distinguish LINH from neoplastic lesions based solely on MRI findings because LINH may transiently show pituitary or pituitary stalk enlargement during the disease course. Frequent MRI in younger children can be invasive because they require sedation during such imaging. Therefore, we believe that anti-rabphilin-3A antibody is more useful as an adjunctive diagnostic marker in younger children.

Glucocorticoids were not administered in this case because of the absence of compressive symptoms associated with the mass lesion, in accordance with the Japan Endocrine Society guidelines. These guidelines recommend glucocorticoid use only when pituitary enlargement causes compressive symptoms (eg, visual defects, severe headaches). Follow-up with pituitary MRI is preferred when symptoms are mild or absent [14]. Additionally, although glucocorticoid treatment has been reported to improve anterior pituitary dysfunction in lymphocytic hypophysitis, AVP-D rarely responds to this therapy [15, 16].

The effective use of anti-rabphilin-3A antibody testing across various age groups may help avoid invasive biopsies for the definitive diagnosis of LINH, and also lead to the establishment of future diagnostic markers and a better understanding of the pathophysiology of LINH. This case reinforces the potential clinical utility of the test as an adjunct in the testing algorithm and should be considered when available. Further accumulation of cases is desirable to support these findings and advance our knowledge of this condition.

## Learning Points

- Differentiating pituitary stalk thickening in children is often challenging.
- Although a pituitary biopsy is necessary to confirm the diagnosis of LINH, anti-rabphilin-3A antibody may serve as an important diagnostic tool.
- The younger the patient, the earlier the regression of pituitary stalk thickening. Combining the measurement of anti-rabphilin-3A antibody with imaging follow-up alone may enhance the likelihood of an early diagnosis.

## Contributors

All authors made individual contributions to authorship. A.Y. and N.K. were involved in the diagnosis and management, obtaining informed consent, and manuscript submission. N.I., H.F., and A.S. were involved in the measurement of anti-rabphilin-3A antibody. Y.S. contributed to reviewing and editing the manuscript. All authors reviewed and approved the final draft.

## Data Availability

Data sharing is not applicable to this article as no datasets were generated or analyzed during the current study.

## Abbreviations

AVP-D: arginine vasopressin deficiency
LINH: lymphocytic infundibuloneurohypophysitis
LPH: lymphocytic panhypopituitaritis
MRI: magnetic resonance imaging
